# Numerical data on the shear stress distribution generated by a rotating rod within a stationary ring over a 35-mm cell culture dish

**DOI:** 10.1016/j.dib.2018.11.087

**Published:** 2018-11-22

**Authors:** Nuttapol Limjeerajarus, Boonrit Keawprachum, Maytus Pliankum, Prasit Pavasant, Chalida Nakalekha Limjeerajarus

**Affiliations:** a Research Center for Advanced Energy Technology, Faculty of Engineering, Thai–Nichi Institute of Technology, Bangkok 10250, Thailand; b Department of Anatomy, Faculty of Dentistry, Chulalongkorn University, Bangkok 10330, Thailand; c Department of Physiology, Faculty of Dentistry, Chulalongkorn University, Bangkok 10330, Thailand; d Excellence Center in Regenerative Dentistry, Faculty of Dentistry, Chulalongkorn University, Bangkok, Thailand

## Abstract

The data contained within this article relate to a rotating rod within a stationary ring that was used to generate shear stress on cells and tissues via a medium. The geometry of the rotating rod within a stationary ring was designed to work with a 35-mm diameter culture dish. The data of the shear stress distribution are presented in terms of area-weighted average shear stress and the uniformity index, which were calculated for medium volumes of 4 and 5 ml at various rotational speeds ranging from 0 to 1000 rpm.

## Specifications table

**Table:** 

Subject area	*Computational Mechanics, Mechanical Engineering*
More specific subject area	*Fluid shear stress, Cell culture*
Type of data	*Figure, Table, numerical value*
How data was acquired	*ANSYS WORKBENCH R16.2 for geometry creation and meshing, and ANSYS-Fluent R16.2 for computational fluid dynamics (CFD) simulation*
Data format	*Raw, analyzed*
Experimental factors	*The properties of the medium were obtained from measurements. The medium density was calculated based on its mass and volume. The medium viscosity was measured using a viscometer (Brookfield, RVDV-I Prime, US).*
Experimental features	*The data of the shear stress distribution at the bottom of a culture dish was simulated based on the CFD technique in ANSYS-Fluent R16.2. The data are presented in terms of area-weighted average shear stress and uniformity index.*
Data source location	*Bangkok, Thailand*
Data accessibility	*The data is with this article*
Related research article	*Charoenpong H, Osathanon T, Pavasant P, Limjeerajarus N, Keawprachum B, Limjeerajarus CN, Cheewinthamrongrod V, Palaga T, Lertchirakarn V and Ritprajak P. Mechanical stress induced human dental pulp S100A7 to augment osteoclast differentiation. Oral Diseases 2018 (*in press*)*[1]

## Value of the data

• The data provide CAD drawing of the rotating rod within a stationary ring that can be used to generate shear stress on cells in a 35-mm diameter culture dish with a uniformity index up to 0.82.
• The uniformity index data can be used by researchers as a benchmark in designing the geometry of a rotating rod or disk for shear stress loading on cells.
• The data present the set up condition of the rotational speed to achieve a desired average shear stress at a specific medium volume.
• Researchers may use the mathematical models obtained from the relationship between the average shear stress and the rotational speed to determine their own desired set up conditions.

## 1 Data

**Fig. 1:**
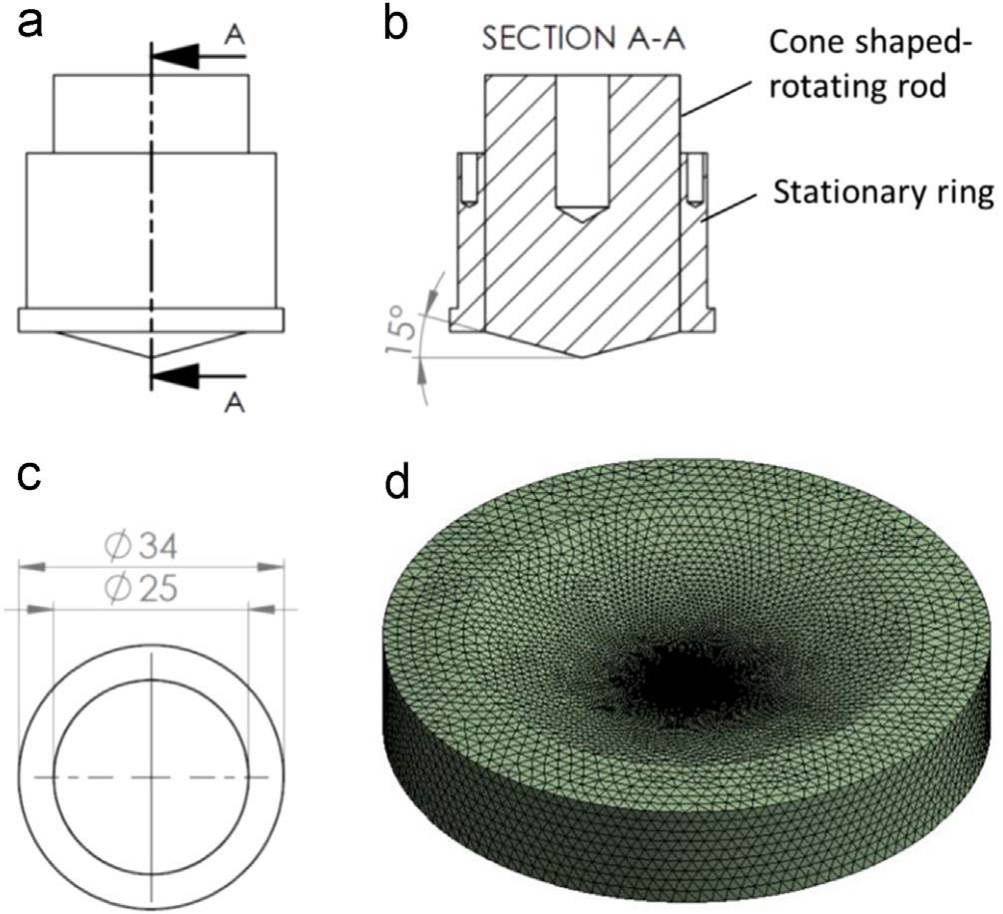
CAD drawing of the rotating rod within the stationary ring; (a) front view, (b) half sectional view, (c) bottom view (dimensions are in mm), and (d) the computational domain used for simulation.

The data presented in this article are based on the numerical simulation of the shear stress distribution generated via a medium by a newly designed rotating rod within a stationary ring that was used to load shear stress on cells and tissue cultured in vitro [1]. The rotating rod within a stationary ring is designed to be used with a 35-mm diameter culture dish. Fig. 1(a)–(c) presents the CAD drawing of the rotating rod within a stationary ring of which the rotating rod is chamfered to be cone-shaped with 15° tilt angle and the stationary ring has inner and outer diameters of 25 and 34 mm, respectively. The rest dimensions in the Fig. 1(a)–(c) are flexible and do not affect the results of the generated shear stress. Fig. 1(d) shows the computational domain of the fluid with polyhedral meshes used in the CFD simulation. The computational domain has an outer diameter of 34 mm as per the diameter at the bottom of the 35-mm diameter culture dish.

**Fig. 2:**
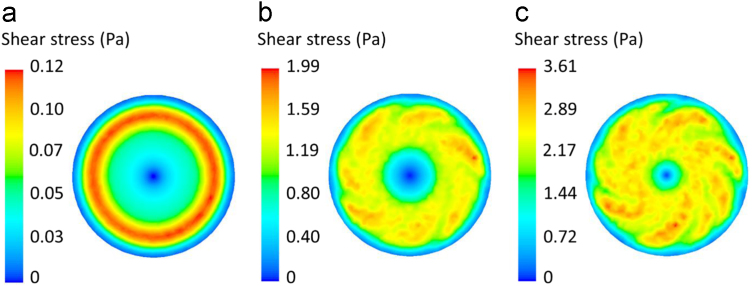
Contour plots of the shear stress distribution at the culture dish bottom when the medium volume is 4 ml at (a) 100, (b) 600, and (c) 1000 rpm.

**Fig. 3:**
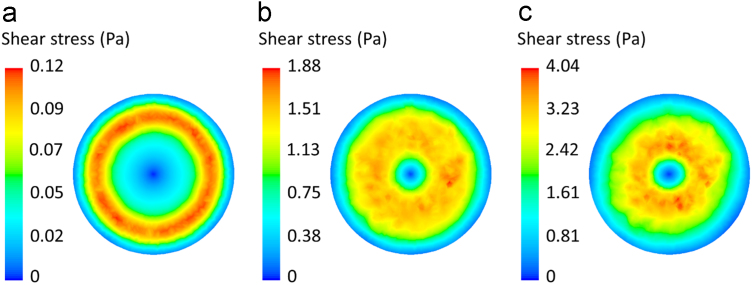
Contour plots of the shear stress distribution at the culture dish bottom when the medium volume is 5 ml at (a) 100, (b) 600, and (c) 1000 rpm.

**Fig. 4:**
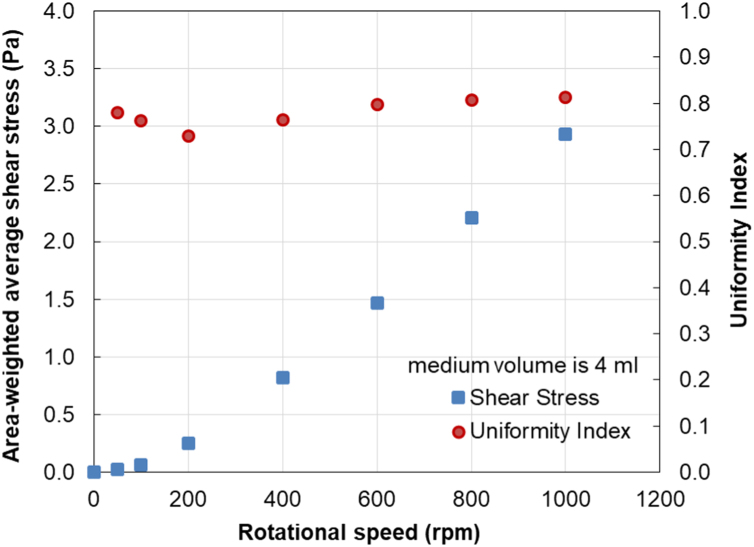
Plots of the area-averaged shear stress and uniformity index at the culture dish bottom when the medium volume is 4 ml.

**Fig. 5:**
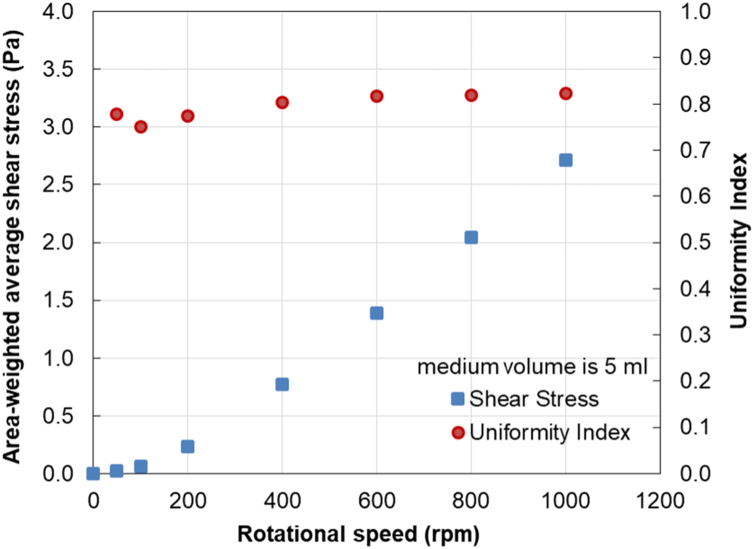
Plots of the area-averaged shear stress and uniformity index at the culture dish bottom when the medium volume is 5 ml.

From simulations, the local shear stress distributions at the culture dish bottom at the rotational speeds of 100, 600 and 1000 rpm for the medium volumes of 4 and 5 ml are depicted in Figs. 2 and 3, respectively. Numerical data of the area-weighted average shear stress and the uniformity index at the bottom of the culture dish generated by the rotating rod within the stationary ring at various rotational speeds are presented in Table 1. These data are also plotted with rotational speeds for the medium volumes of 4 and 5 ml in Figs. 4 and 5, respectively.

**Table 1:** Numerical data of the area-averaged shear stress and uniformity index at the culture dish bottom.

	Medium volume			
	4 ml		5 ml	
Rotational speed	Average shear stress	Uniformity index	Average shear stress	Uniformity index
(rpm)	(Pa)		(Pa)	
0	0.00	–	0.00	–
50	0.02	0.78	0.02	0.78
100	0.06	0.76	0.06	0.75
200	0.25	0.73	0.23	0.77
400	0.82	0.76	0.77	0.80
600	1.47	0.80	1.38	0.82
800	2.21	0.81	2.04	0.82
1000	2.94	0.81	2.71	0.82

For the value of the average shear stress (${\bar{\tau }}_{a}$) at other rotational speeds within a range of 0–1000 rpm, ${\bar{\tau }}_{a}$ can be expressed as a function of rotational speed (*ω*) using the following third-order polynomial equations:

For the medium volume of 4 ml,

(1) $${\bar{\tau }}_{a}=-1.9902\times 10^{-9}\omega^{3}+4.3457\times 10^{-6}\omega^{2}+0.00059221\omega -0.017086$$

For the medium volume of 5 ml,

(2) $${\bar{\tau }}_{a}=-1.8906\times 10^{-9}\omega^{3}+4.0010\times 10^{-6}\omega^{2}+0.00061116\omega -0.018638$$

The standard errors of estimate of the Eqs. (1) and (2) are 0.0164 and 0.0197, respectively.

## 2 Experimental design, materials, and methods

The geometry of the computational domain of the fluid (Fig. 1d) was discretized into computational cells using the finite volume method in the ANSYS WORKBENCH R16.2. The CFD simulation was performed using ANSYS FLUENT R16.2 software to analyze the shear stress distribution at the bottom of the culture dish. The medium temperature was set at 310.15 K. The medium was treated as a Newtonian fluid. The density and viscosity of the medium fluid were 1012.95 kg m^−3^ and 0.00282 kg m^−1^ s^−1^, respectively. The tilt surface of the computational domain of the fluid, which is the cone-shaped rotating rod/medium interface, was selected as the “inlet” surface. The velocity of the inlet surface was input in terms of rad/s. The velocity of the remaining surface of the computational domain was set as zero based on no-slip assumption as it was in contact with either the surface of the stationary ring or the wall surface of the culture dish. The SIMPLE (Semi-Implicit Method for Pressure Linked Equations) algorithm was employed to solve the Navier-Stokes equations iteratively. The calculation in double digit precision was used to achieve simulation results with high accuracy. The second-order upwind discretization scheme was selected to avoid the oscillation of the solution. The under-relaxation factors of the pressure, density, body forces, and momentum were set at 0.27, 1, 1, and 0.55, respectively [2]. The solutions were iterated until the specified convergence criterion of 10^−6^ was achieved. The degree of the model discretization was based on the convergence evaluation results. The final model had 397,307 computational polyhedral cells and 72,510 nodes.

The assumptions made in the simulation were (i) the model was under the steady state and isothermal conditions, (ii) the fluid velocity at a fluid-solid boundary was equal to that of the solid boundary (no-slip condition), (iii) the medium was homogeneous and isotropic, and (iv) the cell height at the bottom of the culture dish was negligible [2].

The criterion used to determine how uniformly the shear stress is distributed over the surface of the culture dish bottom is the area-weighted uniformity index ($\gamma_{a}$), where a value of 1 indicates the highest uniformity. The area-weighted uniformity index can be expressed as [3]:

(3) $$\gamma_{a}=1-\frac{\sum_{i=1}^{n}\left[\left(\left|\tau_{i}-{\bar{\tau }}_{a}\right|A_{i}\right)\right]}{2\left|{\bar{\tau }}_{a}\right|\sum_{i=1}^{n}A_{i}}$$

where, τ is the shear stress (Pa). ${\bar{\tau }}_{a}$ is the area-weighted average shear stress, which is calculated by

(4) $${\bar{\tau }}_{a}=\frac{\sum_{i=1}^{n}\tau_{i}A_{i}}{\sum_{i=1}^{n}A_{i}}$$

The simulations were performed using an Intel Xeon E5-1650 v2 @ 3.5 GHz processor with 32 GB RAM and 4 GB graphic card memory.

## References

[1] Charoenpong H., Osathanon T., Pavasant P., Limjeerajarus N., Keawprachum B., Limjeerajarus C.N., Cheewinthamrongrod V., Palaga T., Lertchirakarn V., Ritprajak P. (2018). Mechanical stress induced human dental pulp S100A7 to augment osteoclast differentiation. Oral Dis..

[2] Keawprachum B., Limjeerajarus N., Limjeerajarus C.N., Srisungsitthisunti P. (2018). Improved design of a cone-shaped rotating disk for shear force loading in a cell culture plate. IOP Conf. Ser.: Mater. Sci. Eng..

[bib3] 3ANSYS^®^ Fluent, Release 16.2, ANSYS Workbench Help, ANSYS, Inc., USA.

