# Supplementary material for: Numerical data on the shear stress distribution generated by a rotating rod within a stationary ring over a 35-mm cell culture dish
Source: Data Brief. 2018 Nov 22;21:2253–8. doi: 10.1016/j.dib.2018.11.087 (PMC6276543; doi:10.1016/j.dib.2018.11.087)
Supplement: Supplementary file 1 — Supplementary material [file mmc1.docx]

**Declarations**

**Author name**:

Chalida Nakalekha Limjeerajarus

**Journal**: Data in Brief

We confirm that the manuscript has been read and approved by all named authors and that there are no other persons who satisfied the criteria for authorship but are not listed. We further confirm that the order of authors listed in the manuscript has been approved by all of us. We confirm that we have given due consideration to the protection of intellectual property associated with this work and that there are no impediments to publication, including the timing of publication, with respect to intellectual property. In so doing we confirm that we have followed the regulations of our institutions concerning intellectual property.

| Authors declared no conflict of interest |
| --- |

**Please state any sources of funding for your research**

| The research leading to these data was funded by the 2012 research chair grant from the National Science and Technology Development Agency (NSTDA), Thailand, and the research grant from the Dental Association of Thailand. The Excellent center in Regenerative Dentistry is supported by the Chulalongkorn Academic Advancement Into Its 2^nd^ Century Project. |
| --- |

**Please state whether Ethical Approval was given, by whom and the relevant Judgement’s reference number**

| none |
| --- |

**If you are submitting a Randomized Controlled Trial, please state the** International Standard Randomised Controlled Trial Number **(ISRCTN)**

| - |
| --- |
